# 570. Evaluating the Association of Frailty with Dermal Substitute Use in Older Adult Burn Patients

**DOI:** 10.1093/jbcr/irag033.310

**Published:** 2026-04-06

**Authors:** Shawn Tejiram, Colette Galet, David M Hill, Lauren T Moffatt, Jeffrey W Shupp, Kathleen S Romanowski

**Affiliations:** MedStar Washington Hospital Center, Washington, DC; University of Iowa, Iowa City, IA; Firefighters Regional One Burn Center, Memphis, TN; The Burn Center at MedStar Washington Hospital Center, Washington, DC; MedStar Washington Hospital Center, Washington, DC; Shriners Children's - Northern California, Sacramento, CA

## Abstract

**Introduction:**

Frailty is established as a predictor of outcomes beyond chronological age and have been used as a measure of physiologic reserve to stratify risk, guide management, and anticipate complications. Older adult burn patients experience unique metabolic and surgical stressors specific to their injury. Dermal substitutes are sometimes used to ameliorate these stressors and delay or minimize definitive autografting. Despite this, literature examining the efficacy of dermal substitute use among frail and non-frail patients is limited. The aim of this study was to compare grafting and related outcomes among frail and non-frail older adult burn injured patients undergoing a dermal substitute surgical management strategy.

**Methods:**

A multicenter trial examining burn injured patients admitted to 12 burn centers from January 2017 to December 2019 and who were 60 years and older were retrospectively reviewed. Demographics, injury characteristics, and clinical metrics were obtained. Frailty was assigned using the Canadian Study of Health and Aging Clinical Frailty Scale (CSHA-CFS) seven point scoring system. Patients were categorized as fit (CFS score < 4), pre-frail (score = 4), and frail (score > 4). Outcomes evaluated included single stage dermal substitute placement vs subsequent autografting, graft loss, length of stay (LOS), in-hospital mortality, and disposition.

**Results:**

Of 1632 older adult burn patients, 805 patients underwent dermal substitute placement. Of these, 386 patients were deemed fit, 203 as pre-frail, and 216 as frail. There was no significant association with the use of dermal substitute with frailty status (*p*=.140) or mortality (*p*=.165). Among institutions that reported, 49 patients underwent subsequent autografting, of which 15 experienced graft loss requiring regrafting. For patients without a dermal substitute, frail patients were more likely to be discharged to a higher level care facility compared to non-frail patients (*p*<.001). Older patients receiving dermal substitutes have significantly higher length of stay [37 (18, 55) vs 13 (6, 21) days, *p*<.001] and discharge to a higher level of care (*p*=.004). Additionally, twice as many frail patients were placed in a higher level care facility as opposed to going home. On multivariable regression, use of a dermal substitute was not associated with placement in a higher level of care facility after controlling for center, burn size, inhalation injury, and frailty (*p*=.511).

**Conclusions:**

While frail patients are more likely to be placed in a higher level of care than return to home, there is not a significant association with use of dermal substitute after controlling for known disposition drivers.

**Applicability of Research to Practice:**

Understanding the association of frailty with dermal substitute strategies in older adult burn patients may influence future management strategies and better anticipate expected outcomes.

**Funding for the study:**

N/A.

